# Fibrin-associated large B-cell lymphoma arising within a hepatic cystic lesion: a case report

**DOI:** 10.3389/fonc.2026.1689561

**Published:** 2026-04-10

**Authors:** Zujun Qin, Yaqi Shu, Qiqi Zhu, Shunhai Jian

**Affiliations:** Department of Pathology, Affiliated Hospital of North Sichuan Medical College, Nanchong, Sichuan, China

**Keywords:** B-cell, Epstein-Barr virus, fibrin-associated, liver, lymphoma

## Abstract

Fibrin-associated large B-cell lymphoma (FA-LBCL) is a rare Epstein-Barr virus (EBV)-associated B-cell neoplasm typically arises within chronic fibrin deposits in confined anatomical spaces, whether natural or acquired. It is most commonly reported in association with cardiac myxomas, vascular prostheses and organizing hematomas. Here, we describe the first documented case of FA-LBCL arising within a hepatic cystic lesion. A 69-year-old immunocompetent man was found incidentally to have a large cystic mass in the right hepatic lobe without contrast enhancement on imaging. The lesion was surgically resected. Histologic examination revealed scattered aggregates of large atypical B lymphocytes embedded in abundant fibrin and necrotic material without invasive growth. Tumour cells were positive for pan-B cell markers CD20 and CD79α, exhibited a MUM1 index of approximately 50%, demonstrated a high proliferative index, and tested positive for Epstein-Barr virus-encoded RNA (EBER) by *in situ* hybridization, supporting the diagnosis of FA-LBCL. The patient received no additional treatment and remained recurrence-free at 9 months’ follow-up. This report reviews the literature on FA-LBCL, emphasizing clinicopathologic features, proposed pathogenesis, and key differential diagnoses. This case broadens the anatomic spectrum of FA-LBCL, and emphasises the need for heightened awareness of this condition when it occurs in uncommon sites to avoid misdiagnosis.

## 1 Introduction

FA-LBCL is formally recognized as a distinct entity in the 5th edition of the World Health Organization (WHO) Classification of Haematolymphoid Tumours. During the evolution of disease classification, FA-LBCL was previously categorized under diffuse large B-cell lymphoma (DLBCL), specifically regarded as a subtype of DLBCL associated with chronic inflammation (DLBCL-CI) (1). However, unlike conventional DLBCL, FA-LBCL typically does not form invasive masses and rarely causes clinical symptoms. It is often incidentally discovered during histological examination of surgical specimens removed for other reasons. Previous studies indicate that this disease predominantly follows an indolent clinical course, with favorable prognosis following simple surgical resection.

FA-LBCL is thought to originate in anatomical sites with chronic fibrin deposition. Reported cases predominantly occur in the cardiovascular system, including lesions associated with cardiac myxomas, prosthetic implants, or vascular aneurysms (2). However, chronic fibrin deposition may also be observed in other anatomical sites, particularly within certain cystic lesions. In recent years, a small number of cases have been reported involving FA-LBCL in organs such as the ovary, thyroid, adrenal gland and spleen (3–6). To our knowledge, this case represents the first reported instance of FA-LBCL arising within a hepatic cystic lesion.

## 2 Case presentation

**Figure 1:**
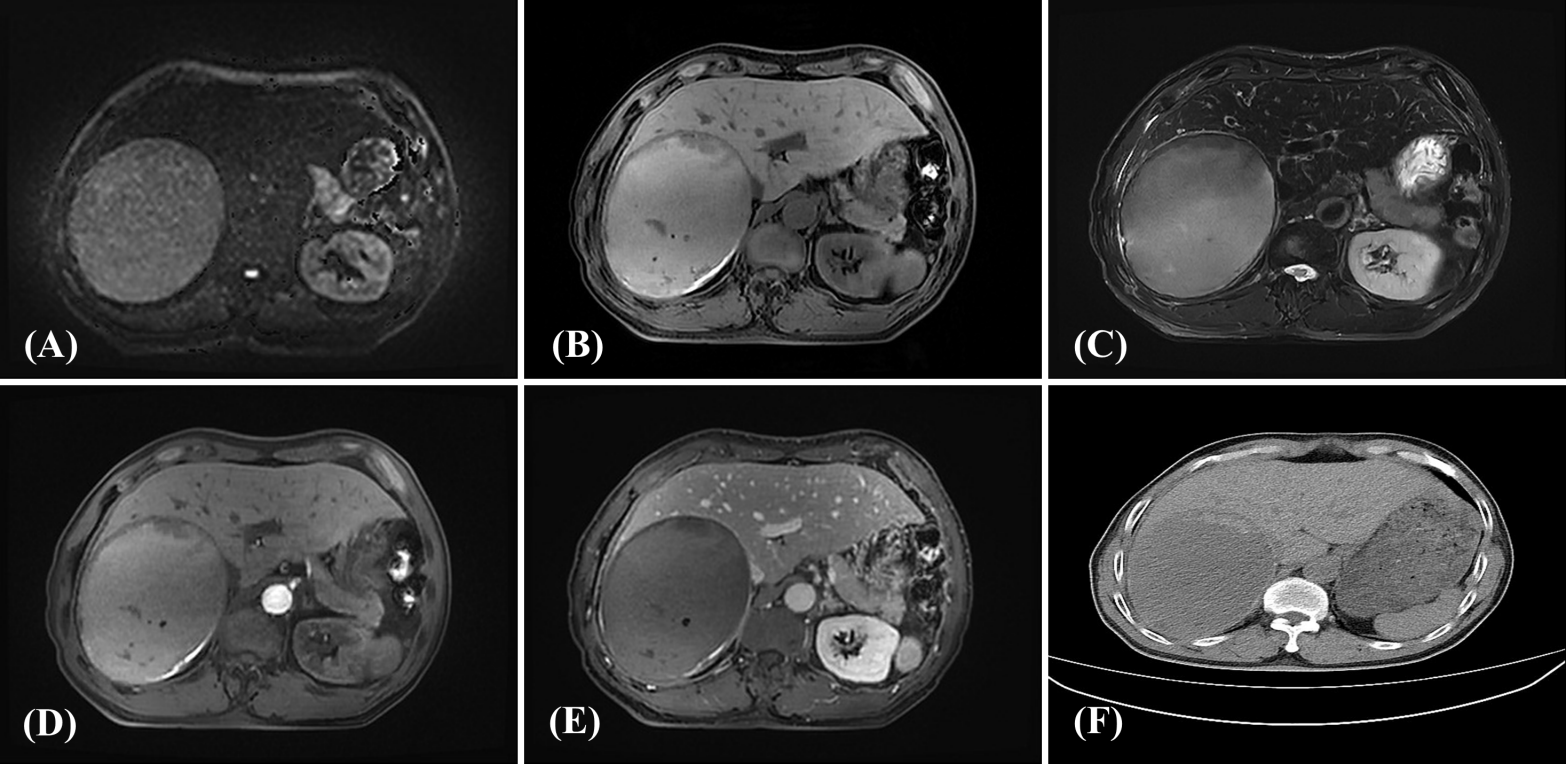
DWI demonstrated no appreciable restricted diffusion **(A)**. The lesion appeared isointense to mildly hyperintense on T1-weighted imaging (T1WI) **(B)**. The lesion demonstrated hyperintense signal on T2-weighted fat-suppressed imaging (FS-T2WI) **(C)**. No appreciable enhancement was observed during the arterial phase of contrast-enhanced scanning **(D)**. No appreciable enhancement was observed during the portal venous phase of contrast-enhanced scanning **(E)**. CT imaging demonstrated a mildly hypodense mass in the posterior segment of the right hepatic lobe **(F)**.

A 69-year-old immunocompetent male presented with an incidentally detected hepatic lesion on screening computed tomography (CT). Initial CT demonstrated a 14.5 cm space-occupying lesion with mildly hypodense attenuation in the right hepatic lobe, radiologically suggestive of concurrent multiple hepatic hemangiomas. Subsequent contrast-enhanced magnetic resonance imaging (CE-MRI) further characterized a large mass (11.8 × 13.0 × 11.8 cm) in the right hepatic lobe, demonstrating well-defined margins with an identifiable pseudocapsule and heterogeneous signal intensity (isointense to mildly hyperintense on T1- and T2-weighted imaging with scattered cystic foci exhibiting prolonged T1 and T2 relaxation times). The lesion exhibited no significant restricted diffusion on high-b-value diffusion-weighted imaging (DWI) and no appreciable enhancement on post-contrast sequences **Figure 1**. Significant mass effect caused compression of the inferior vena cava (IVC) and inferior displacement of the right kidney. Additional findings included multiple enhancing nodules within the hepatic parenchyma (largest 0.7 cm), demonstrating restricted diffusion and a characteristic hemangioma enhancement pattern (rapid arterial phase enhancement with persistent delayed enhancement), along with scattered small non-enhancing cysts.

The patient underwent left-hand vascular rupture surgical repair at a local hospital three years ago (details unclear). He reported a smoking history of over 50 years, approximately 1 pack per day, and an alcohol consumption history of over 50 years, approximately 100 mL per day. No clinical history of autoimmune diseases was documented.

Gross examination revealed a gray-red hepatic specimen measuring 19.5 × 17 × 7.3 cm. Multiple sections were cut, revealing a large mass approximately 14.5 × 14 × 7.3 cm. The cut surface is cystic, with the cyst cavity containing many gray-yellow to gray-brown, chocolate-colored gelatinous material. The cyst wall is well-defined from the surrounding tissue and is close to the liver capsule.

**Figure 2:**
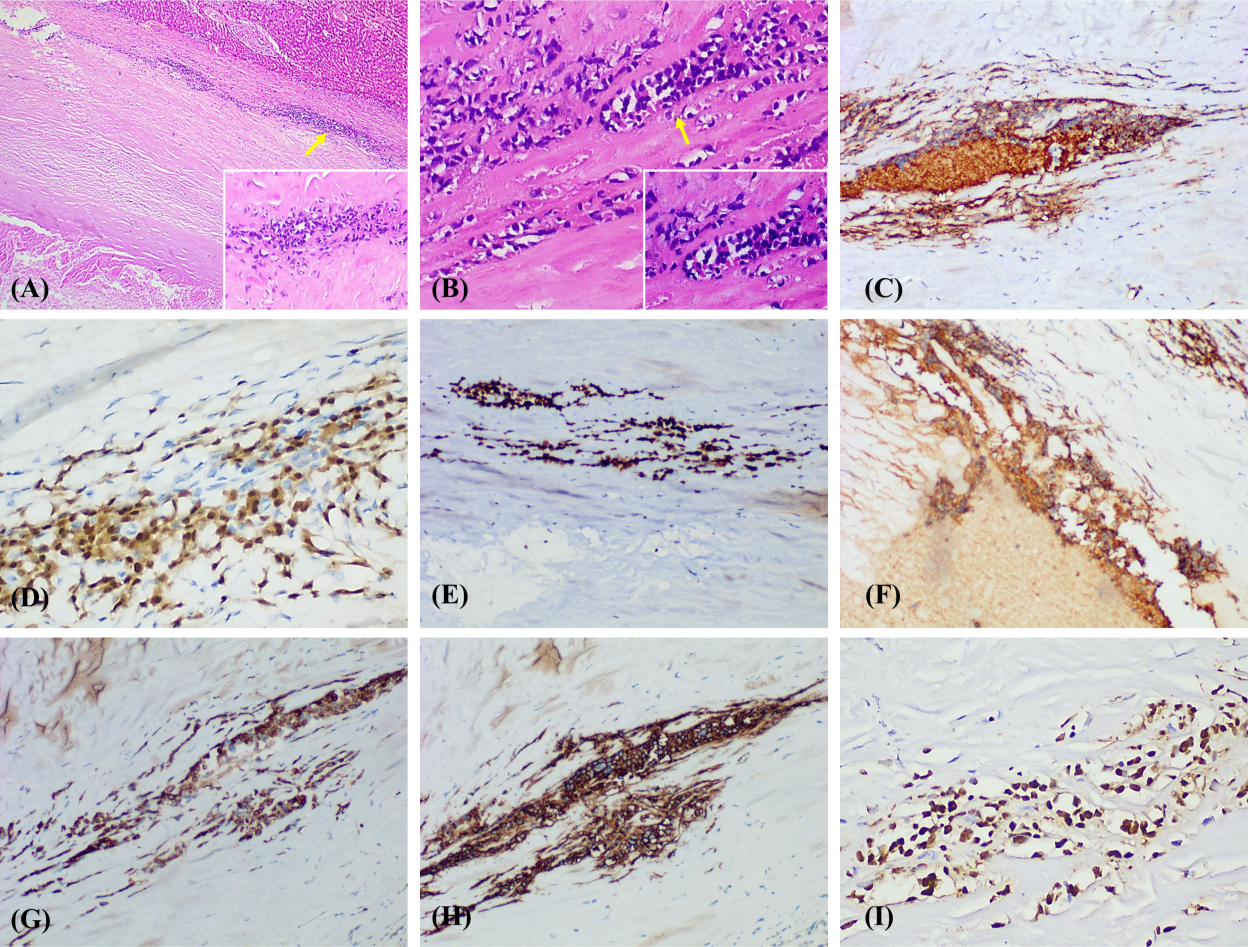
**(A, B)** Histologic examination (H&E) revealed fibrous tissue proliferation in the pseudocapsule, with scattered lymphocyte clusters infiltrating and abundant necrosis. High-power fields show atypical lymphocytes with nuclear diameters exceeding twice that of small lymphocytes, with cells notably enlarged, pleomorphic, and showing coarse chromatin. Pathological mitosis is visible. [**(A)** Scale bar = 500 μm, 4× and 100 μm, 20×; **(B)** Scale bar = 100 μm, 20× and 50 μm, 40×]. Immunohistochemistry confirmed B-cell origin: CD45+[**(C)**,20×] CD20+ [**(H)**, 20×], CD79α+ [**(G)**, 20×], CD38+ [**(F)**, 20×], and Ki-67 index was about 80% [**(E)**, 20×], MUM1 index was about 50% [**(D)**,40×]. Additionally, Tumour cells were positive for EBV-encoded RNA (EBER) *in situ* hybridization [**(I)**,40×].

**Figure 3:**
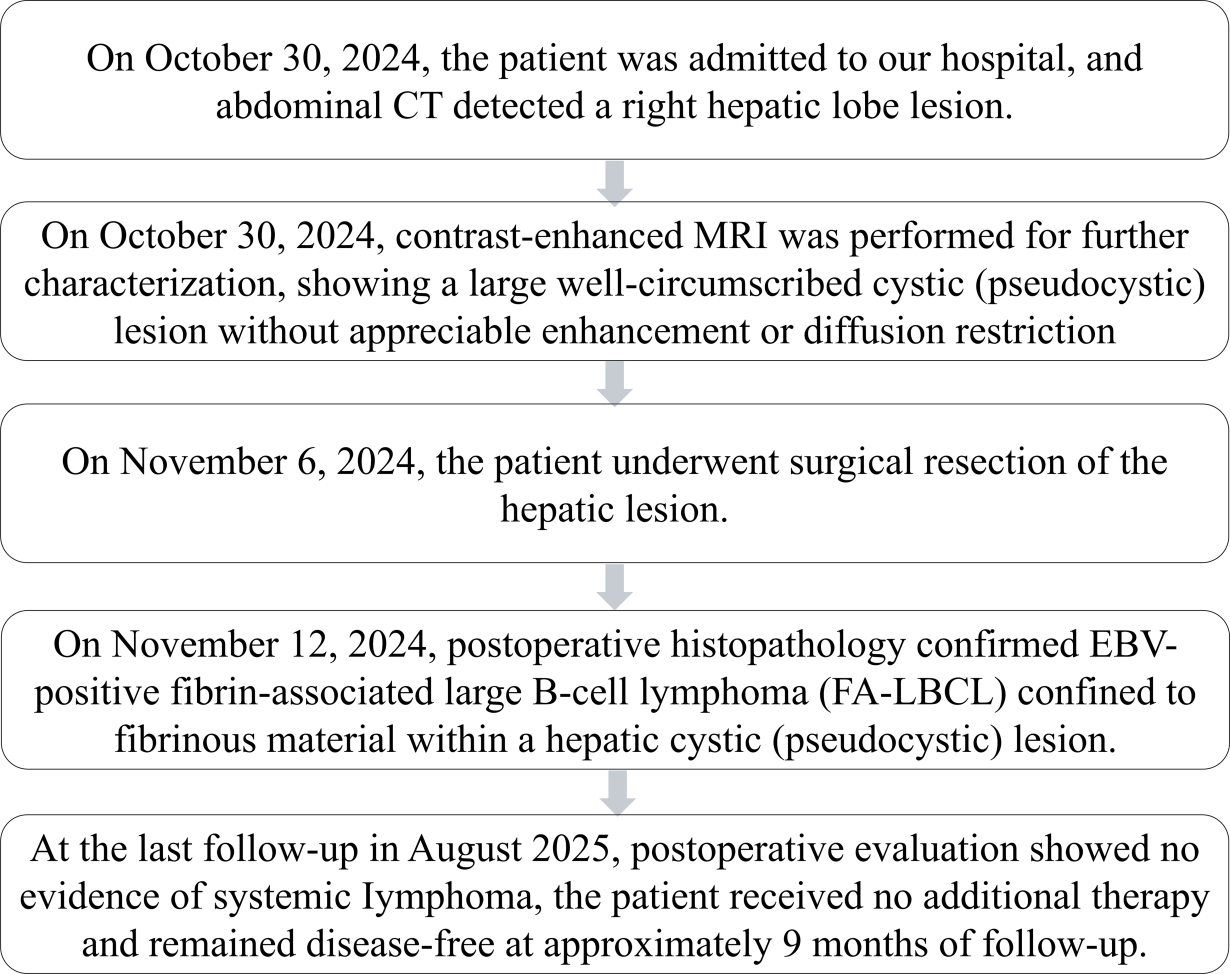
Timeline of this case.

Combined analysis of histomorphology and immunohistochemistry (IHC) supported the diagnosis of FA-LBCL in the right hepatic lobe **Figure 2**. Further physical examination and imaging studies revealed no evidence of extrahepatic involvement. Comprehensive clinical evaluation and imaging studies revealed no evidence of lymphadenopathy or extranodal disease. PET-CT and bone marrow biopsy were not performed due to the absence of clinical or radiologic suspicion of systemic lymphoma. The patient has not received any treatment other than surgery. During 9 months of follow-up since diagnosis, no evidence of recurrence has been observed. The timeline of this case is depicted in **Figure 3**.

The Medical Ethics Committee of the Affiliated Hospital of North Sichuan Medical College approved this study. Clinical data, including symptoms, medical history, laboratory tests, imaging studies, treatment, and follow-up, were all obtained from the medical records. The specimens were fixed in 10% buffered formalin for 24 hours, followed by routine processing, paraffin embedding, and hematoxylin-eosin (H&E) staining.

## 3 Discussion

Fibrin-associated large B-cell lymphoma (FA-LBCL) is an uncommon subtype of diffuse large B-cell lymphoma associated with chronic inflammation. In the fifth edition of the World Health Organization (WHO) classification, the term “FA-DLBCL” was revised to “FA-LBCL” for standardization purposes, as its diffuse growth pattern is not prominent (7). Furthermore, FA-LBCL, with its indolent clinical course, is now distinguished from chronic inflammation-associated diffuse large B-cell lymphoma (DLBCL-CI), which is classified as an aggressive tumour and no longer included in that category. FA-LBCL is non-invasive and occurs in localized chronic fibrin deposition, making it particularly prominent in pseudocystic lesions. It is associated with conditions such as pseudocysts, atrial myxomas, valve prostheses, fibrin thrombi, synthetic vascular grafts, hydrocele, metal implants, and chronic subdural hematomas (4).

From a radiologic perspective, hemangioma was initially considered due to the presence of multiple smaller lesions demonstrating typical enhancement patterns; however, the large lesion lacked the characteristic peripheral nodular enhancement and showed no progressive centripetal fill-in. Simple or hemorrhagic cysts were less favored due to the presence of a pseudocapsule and heterogeneous internal contents. The absence of contrast enhancement and restricted diffusion further argued against a solid malignant neoplasm. These imaging findings made preoperative diagnosis challenging, in FA-LBCL and explain why the lesion was not suspected to be lymphoid in origin. The main differential diagnoses included cavernous hemangioma, complex hepatic cyst, and cystic neoplasm.

FA-LBCL exhibits relatively characteristic histopathological features, typically presenting as cystic or pseudocystic structures with abundant fibrin deposition visible microscopically. Within the fibrinous matrix, large atypical B lymphocytes aggregate intermittently, often forming band-like or patchy infiltrates against a background of fibrin and necrotic material. These infiltrates do not invade adjacent tissue structures, reflecting the tumour’s non-invasive growth pattern. Immunophenotypically, FA-LBCL tumour cells originate from B cells. Nearly all previously reported cases exhibit MUM1/IRF4 positivity and CD10 negativity, supporting a non-germinal center B-cell (non-GCB) immunophenotype (5). Concurrently, Ki-67 often indicates high proliferative activity. In this case, immunohistochemistry revealed positivity for CD20, CD79α, CD45, and CD38. The Ki-67 proliferation index was approximately 80%, and the MUM1 index was approximately 50%, *in situ* hybridization for EBV-encoded RNA (EBER) was positive. In addition, they were negative for CD3, CK, CK-H, CK-L, CD56, CD34, Syn, CgA. These morphological and immunophenotypic features align with the typical presentation of FA-LBCL.

Most cases have a strong association with EBV infection. However, there are a few reports of EBV-negative FA-LBCL cases (8). EBV infection of type III latency is typically a hallmark of lymphoproliferative disorders occurring under conditions of severe immunosuppression. However, most cases of FA-LBCL occur in immunocompetent individuals (9). The pathogenesis of FA-LBCL remains unclear, but several potential pathogenic mechanisms have been proposed.

Most evidence suggests that its “initiation/growth substrate” is closely associated with fibrin (and necrotic) deposition within confined anatomical spaces, rather than being caused by systemic immunodeficiency. Some researchers speculate that such fibrin clots often exhibit poor blood supply and restricted access for immune cells, potentially forming a relatively “immunologically isolated” microenvironment. This environment may enable latent EBV-infected B cells to evade T-cell surveillance more readily locally and gain opportunities for clonal expansion (10). Based on the common locations of its occurrence, it is speculated that FA-LBCL may be related to the presence of foreign bodies, chronic inflammation, and the duration of these conditions. These factors may promote the formation and persistence of such fibrin-rich microenvironments. We have observed that its onset is always associated with some underlying lesion, whether natural (such as pseudocysts, atrial myxomas, organizing hematomas, etc.) or artificial (such as metal implants, breast prostheses, valve prostheses, etc.). In the present case, although no prior hepatic disease was documented, the patient had a prolonged history of heavy alcohol consumption. It is known that long-term alcohol consumption induces chronic inflammatory changes leading to chronic alcoholic liver injury (11). Although a direct causal relationship cannot be established, prolonged alcohol exposure may promote a local inflammatory environment, facilitating fibrin accumulation within cystic lesions.

Given the localized, non-invasive nature of FA-LBCL, complete surgical resection is generally considered sufficient, particularly for fully resectable lesions which exhibit low recurrence rates. Systemic therapy is not routinely administered in the absence of disseminated disease. In contrast, chemotherapy may increase the risk of death due to its associated complications (4). A study published by Daniel F. Boyer et al. (12) in 2023 indicated that the disease is highly associated with PTEN inactivation mutations. When the lesion cannot be completely resected, inhibition of the PI3K/AKT pathway may be a potential treatment option for FA-LBCL.

## 4 Differential diagnosis

a. Diffuse large B-cell lymphoma associated with chronic inflammation (DLBCL-CI): DLBCL-CI is a rare EBV-positive lymphoma typically occurring in immunocompetent patients and associated with long-standing chronic inflammation, such as pyothorax, chronic osteomyelitis, metallic implant, or chronic skin ulcers. Pyothorax-associated lymphoma (PAL) is the most common clinical presentation, accounting for approximately 80% of all DLBCL-CI cases (13). Although DLBCL-CI shares morphological similarities with FA-LBCL—including large B-cell morphology, B-cell immunophenotype, high proliferation index, and EBV association—they exhibit distinct differences in aggressive features. Tumour cells in DLBCL-CI typically exhibit diffuse invasive growth, frequently destroying adjacent tissues and accompanied by marked acute and chronic inflammatory cell infiltration. DLBCL-CI predominantly affects elderly patients and often has a prolonged latency period, despite systemic chemotherapy, the five-year survival rate remains low (approximately 22%) (14). Distinguishing DLBCL-CI from FA-LBCL histologically is challenging. DLBCL-CI typically presents as focal infiltration of preexisting tissue or as a mass lesion, whereas FA-LBCL is frequently associated with fibrin deposition, which is less common in DLBCL-CI. Differential diagnosis requires consideration of the patient’s clinical characteristics, the aggressiveness of the mass, and prognostic factors to aid in distinguishing between the two.

b. Primary effusion lymphoma (PEL) typically occurs in the pleural cavity and is driven by human herpesvirus 8 (HHV8). The tumour cells are suspended in the effusion fluid without solid mass formation. PEL tumour cells usually do not express “pan-B-cell” markers, including CD19, CD79α, and CD20. They may express varying degrees of markers common to terminally differentiated plasma B cells, including CD38, CD138, MUM1, and EMA, but do not express the marker PAX5 (15). Combining clinical history and immunohistochemical profile allows clear differentiation from FA-LBCL.

c. Lymphomatoid granulomatosis (LYG): This rare EBV-associated lymphoproliferative disorder primarily involves the lungs (16). It is characterized by angiocentricity and angiodestruction, leading to varying degrees of coagulative necrosis. LYG is frequently associated with immunodeficiency (17) and has no history of long-term chronic inflammation. The lesions consist of EBV-positive B cells and reactive T cells, with reactive T cells usually being the predominant component. The lesions often express a large number of T-cell markers. The disease can be classified into grades 1–3 based on the number of EBV-positive large B cells. High-grade (grade 3) LYG requires combined immunochemotherapy, while low-grade (grade 1 and 2) LYG can be treated with immune enhancers or monitored for observation.

## 5 Conclusions

Fibrin-associated large B-cell lymphoma (FA-LBCL) is a rare EBV-associated lymphoproliferative disorder that occurs within fibrinous material in various clinical contexts. Hepatic involvement is extremely rare, and this report describes the first documented hepatic occurrence of FA-LBCL identified in association with a hepatic cystic lesion. The disease is characteristically devoid of mass formation, with only minute tumour foci present, posing significant diagnostic challenges. The number of reported FA-LBCL cases is still limited, and evidence-based treatment guidelines remain undefined. Additional cases with long-term follow-up are needed to better define optimal management strategies. Here, we report the first case of hepatic occurrence of FA-LBCL identified in association with a hepatic cystic lesion, which expands the known clinicopathological spectrum of FA-LBCL and provides new insights into its pathological diagnosis and treatment strategies.

## Data Availability

The original contributions presented in the study are included in the article/supplementary material. Further inquiries can be directed to the corresponding author.

## References

[1] WHO Classification of Tumours Editorial Board, Cree IA, Moch H, editors . Haematolymphoid tumours. 5th ed. Vol. 11. Lyon: IARC Publications. (2024). Available online at: https://www.zora.uzh.ch/id/eprint/268312/ (Accessed March 31, 2026).

[2] Chu W Zhang B Zhang Y Tian D Tang Y Zhang W . Fibrin-associate diffuse large B-cell lymphoma arising in a left atrial myxoma: a case report and literature review. Cardiovasc Pathol. (2020) 49:107264. doi: 10.1016/j.carpath.2020.107264. PMID: 32805552

[3] Ho TW Cheuk W Chan JKC . EBV-negative fibrin-associated large B-cell lymphoma arising in thyroid hyperplastic nodule: Report of a case and literature review. Int J Surg Pathol. (2023) 31:1420–5. doi: 10.1177/10668969231152586. PMID: 36843554

[4] Kircher S Böck J Maurus K Düll J Topp M Seitz AK . Fibrin-associated large B-cell lymphoma arising in a cystic lymphangiomatous lesion of the adrenal gland: a case report and overview of the entity. Pathol Res Pract. (2025) 270:155957. doi: 10.1016/j.prp.2025.155957. PMID: 40215669

[5] Louro LS Miranda RN Medeiros LJ Malpica A Marques-Piubelli ML Ramalingam P . From the archives of MD anderson cancer center: EBV-positive fibrin-associated large B-cell lymphoma in an ovarian leiomyoma with cystic degeneration: a case report and discussion of differential diagnosis. Ann Diagn Pathol. (2025) 74:152397. doi: 10.1016/j.anndiagpath.2024.152397. PMID: 39608291

[6] Justo I Jiménez-Romero C Suárez A Vazquez P Revilla E Loinaz C . Splenic cyst deroofing complicated with B lymphoma. World J Surg Oncol. (2024) 22:231. doi: 10.1186/s12957-024-03509-z. PMID: 39232740 PMC11373119

[7] Pak MG Roh MS . Fibrin-associated large B-cell lymphoma arising in an endovascular graft: first case report in korea. J Pathol Transl Med. (2024) 58:87–90. doi: 10.4132/jptm.2023.12.28. PMID: 38253476 PMC10948249

[8] Baugh L Brown N Song JY Pandya S Montoya V Perry AM . Fibrin-associated, EBV-negative diffuse large B-cell lymphoma arising in atrial myxoma: Expanding the spectrum of the entity. Int J Surg Pathol. (2022) 30:39–45. doi: 10.1177/10668969211014959. PMID: 33913371

[9] Zanelli M Zizzo M Montanaro M Gomes V Martino G De Marco L . Fibrin-associated large B-cell lymphoma: First case report within a cerebral artery aneurysm and literature review. BMC Cancer. (2019) 19:916. doi: 10.1186/s12885-019-6123-1. PMID: 31519155 PMC6743119

[10] Gralewski J Babu D . Diffuse large B-cell lymphoma associated with chronic inflammation and fibrin-associated large B-cell lymphoma. In: Crane GM Loghavi S , editors.Precision Molecular Pathology of Aggressive B-cell Lymphomas. Springer International Publishing, Cham (2023). p. 339–50. doi: 10.1007/978-3-031-46842-1_21, PMID:

[11] Lieber CS . Alcohol: its metabolism and interaction with nutrients. Annu Rev Nutr. (2000) 20:395–430. doi: 10.1146/annurev.nutr.20.1.395. PMID: 10940340

[12] Boyer DF Perry A Wey E Hsueh J Li A Jackson R . Fibrin-associated large B-cell lymphoma shows frequent mutations related to immune surveillance and PTEN. Blood. (2023) 142:1022–5. doi: 10.1182/blood.2023020349. PMID: 37433264 PMC10517201

[13] Martin de Bustamante JM Mendoza A López-Muñoz S García-Fernández E Gómez-Prieto P Jiménez-Yuste V . A new face of fibrin-associated large B-cell lymphoma: epstein–barr virus-positive breast implant-associated diffuse large B-cell lymphoma. J Clin Med. (2023) 12:3614. doi: 10.3390/jcm12113614. PMID: 37297811 PMC10253260

[14] Beltran BE Castro D Paredes S Miranda RN Castillo JJ . EBV-positive diffuse large B-cell lymphoma, not otherwise specified: 2020 update on diagnosis, risk-stratification and management. Am J Hematol. (2020) 95:435–45. doi: 10.1002/ajh.25760. PMID: 32072672

[15] Brumbaugh B Sugden B . A critical role for epstein-barr virus in primary effusion lymphoma. Berlin, Heidelberg: Springer (2025) p. 1–20. doi: 10.1007/82_2025_310, PMID: PMC12851538 40423780

[16] Taguchi R Tanaka Y Shimmura M Takano H Kakehashi A Kaburaki T . Multiple choroidal granulomas in lymphomatoid granulomatosis: a case report. BMC Ophthalmol. (2025) 25:223. doi: 10.1186/s12886-025-04030-x. PMID: 40251571 PMC12007125

[17] Melani C Jaffe ES Wilson WH . Pathobiology and treatment of lymphomatoid granulomatosis, a rare EBV-driven disorder. Blood. (2020) 135:1344–52. doi: 10.1182/blood.2019000933. PMID: 32107539 PMC7162687

